# Supplementary figures and images for: Is there a link between aging and microbiome diversity in exceptional mammalian longevity?
Source: PeerJ. 2018 Jan 8;6:e4174. doi: 10.7717/peerj.4174 (PMC5764031; doi:10.7717/peerj.4174)

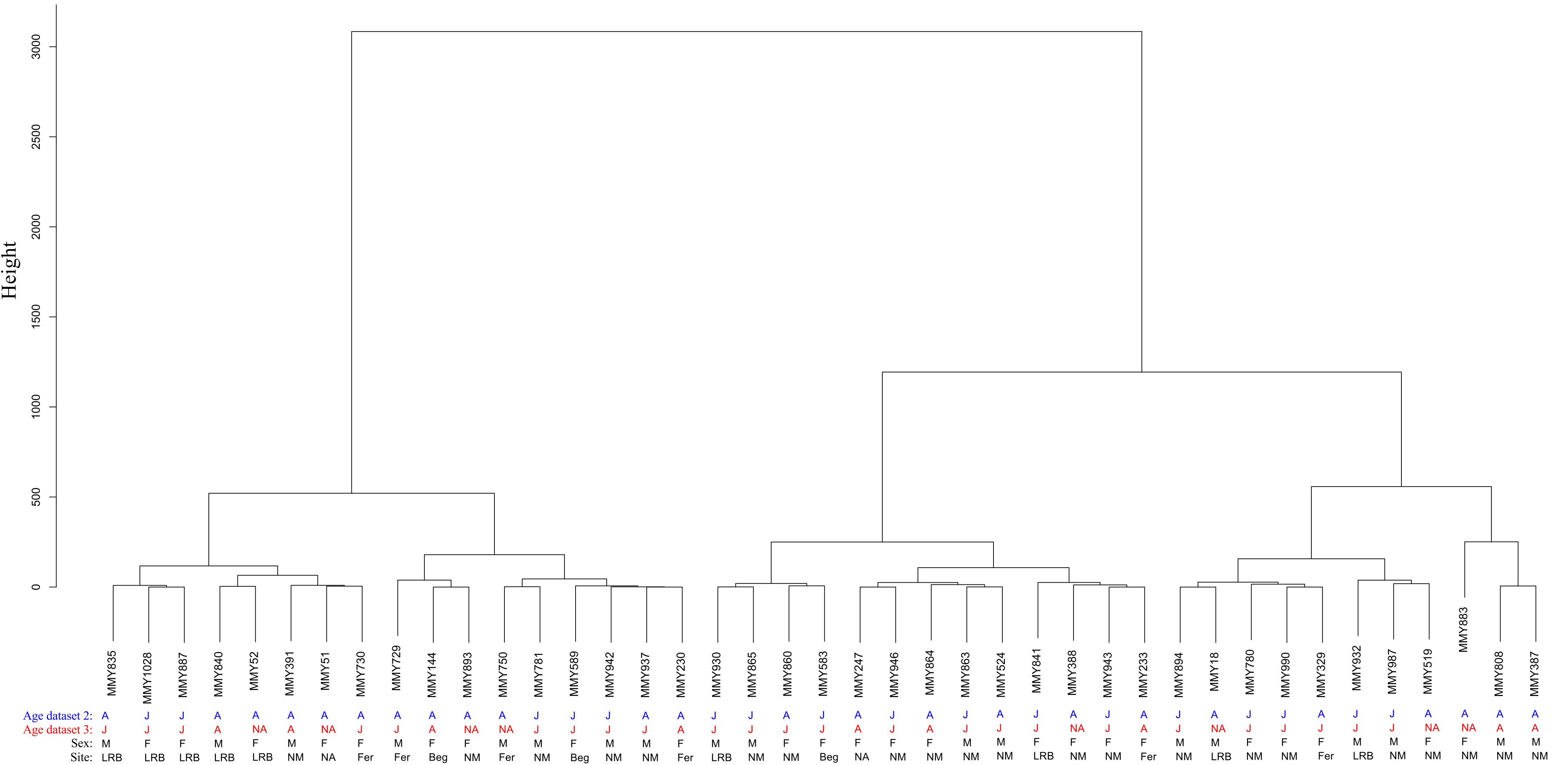

Supplement: Figure S1 — Myotis myotis samples were grouped using hierarchical clustering based on the number of different OTUs found across each individual. [file peerj-06-4174-s001.pdf]

Age Dataset 1

(a)

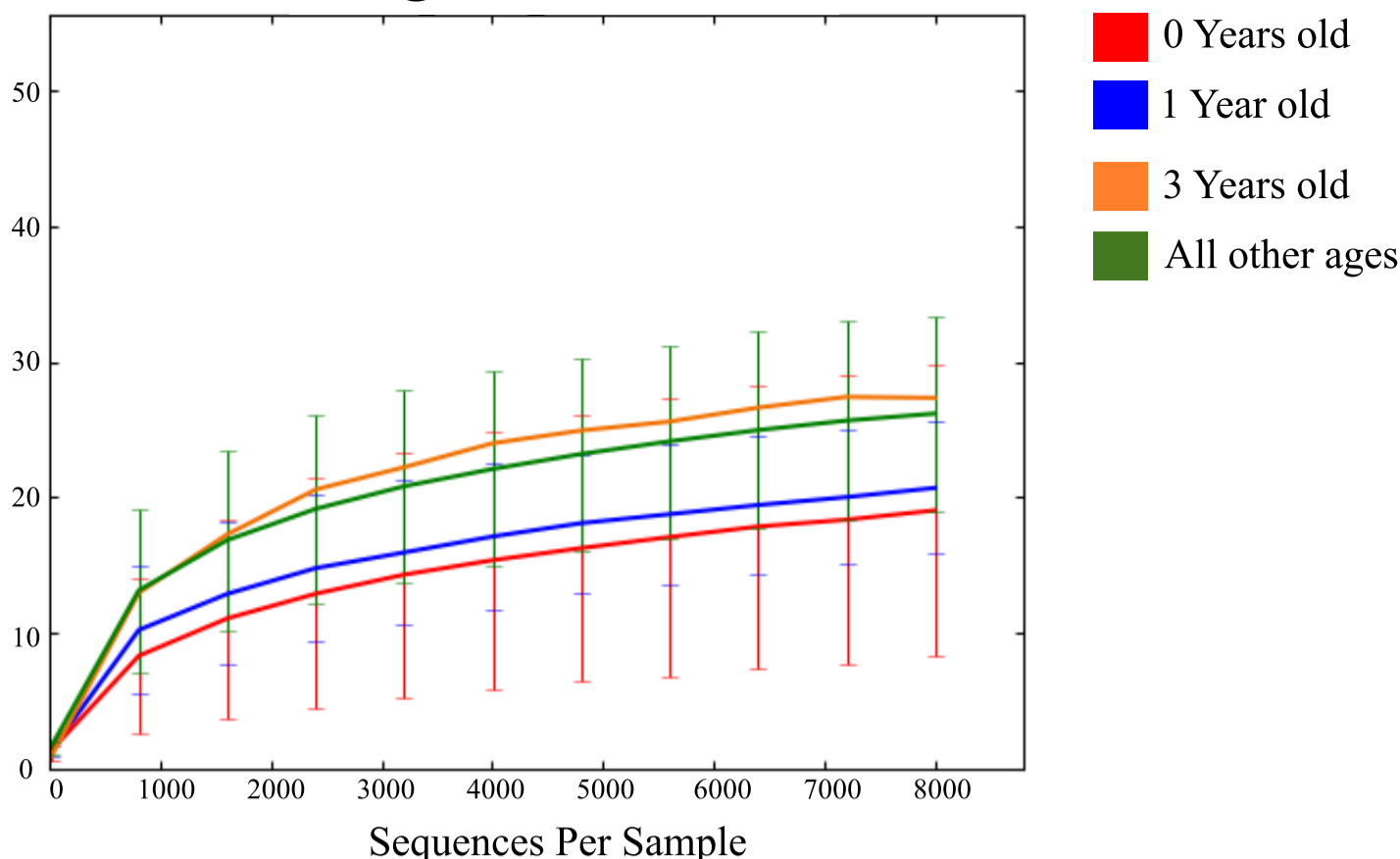

Age Dataset 3

(b)

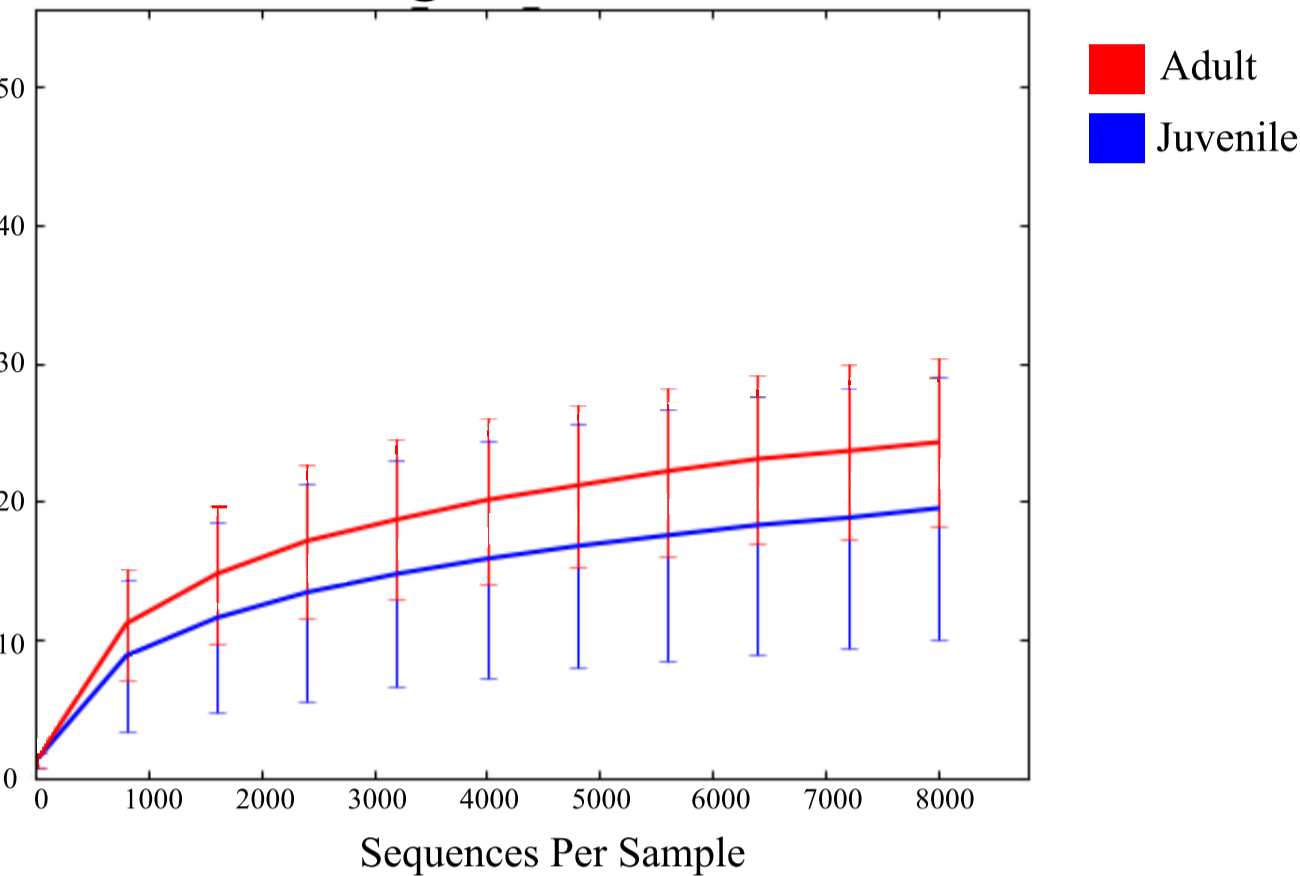

Age Dataset 4

(c)

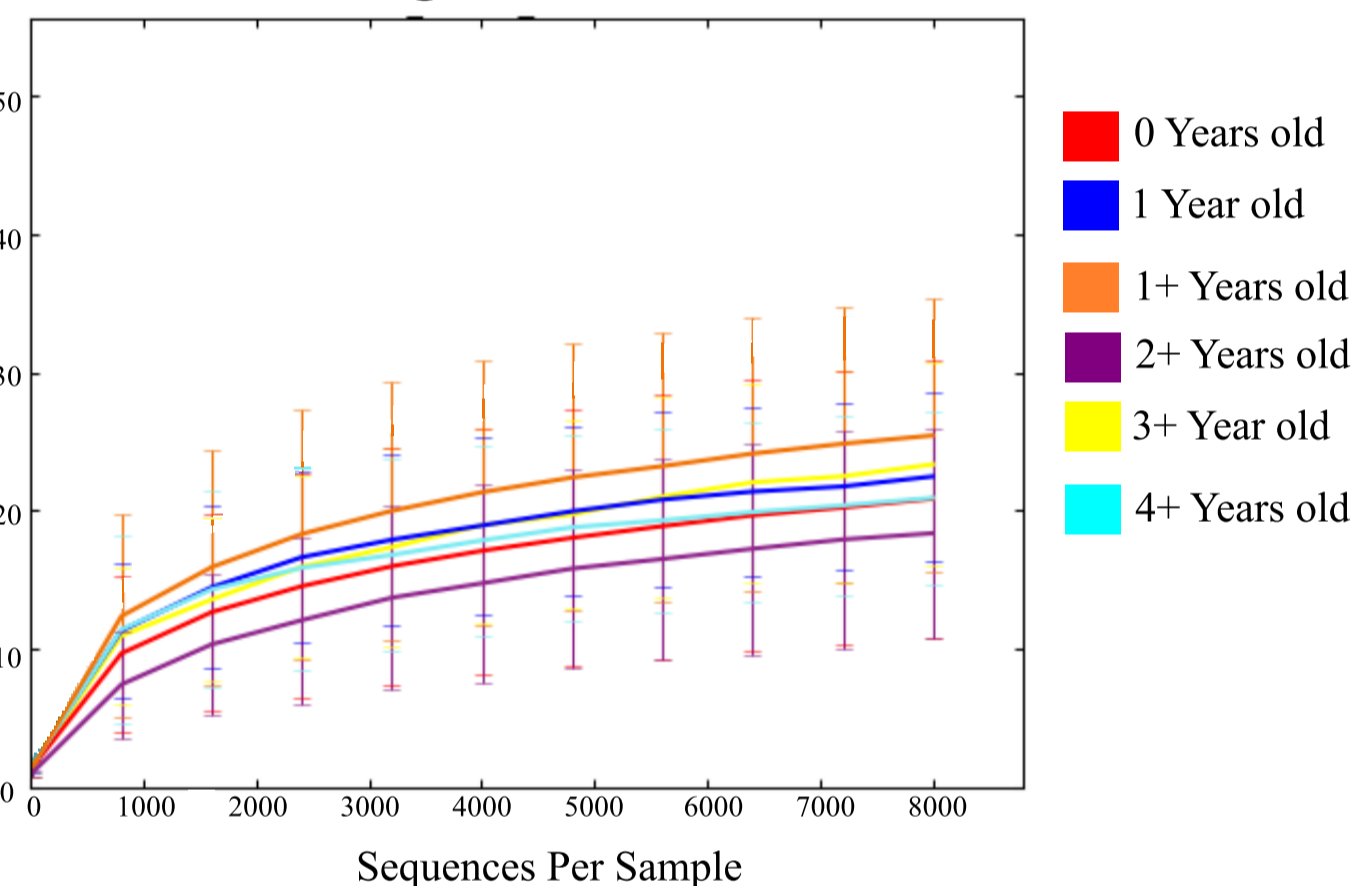

Site

(d)

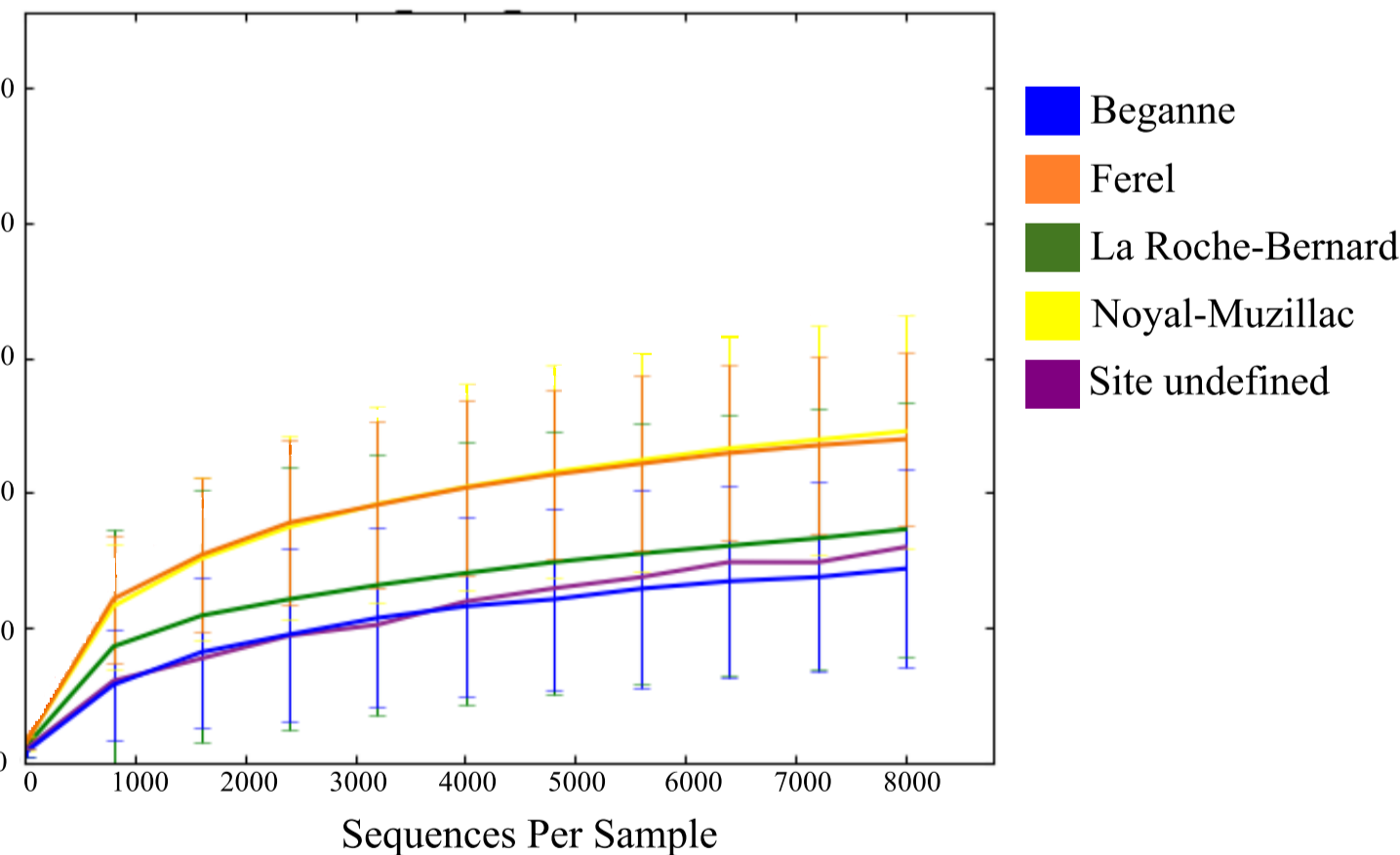

Sample ID

(e)

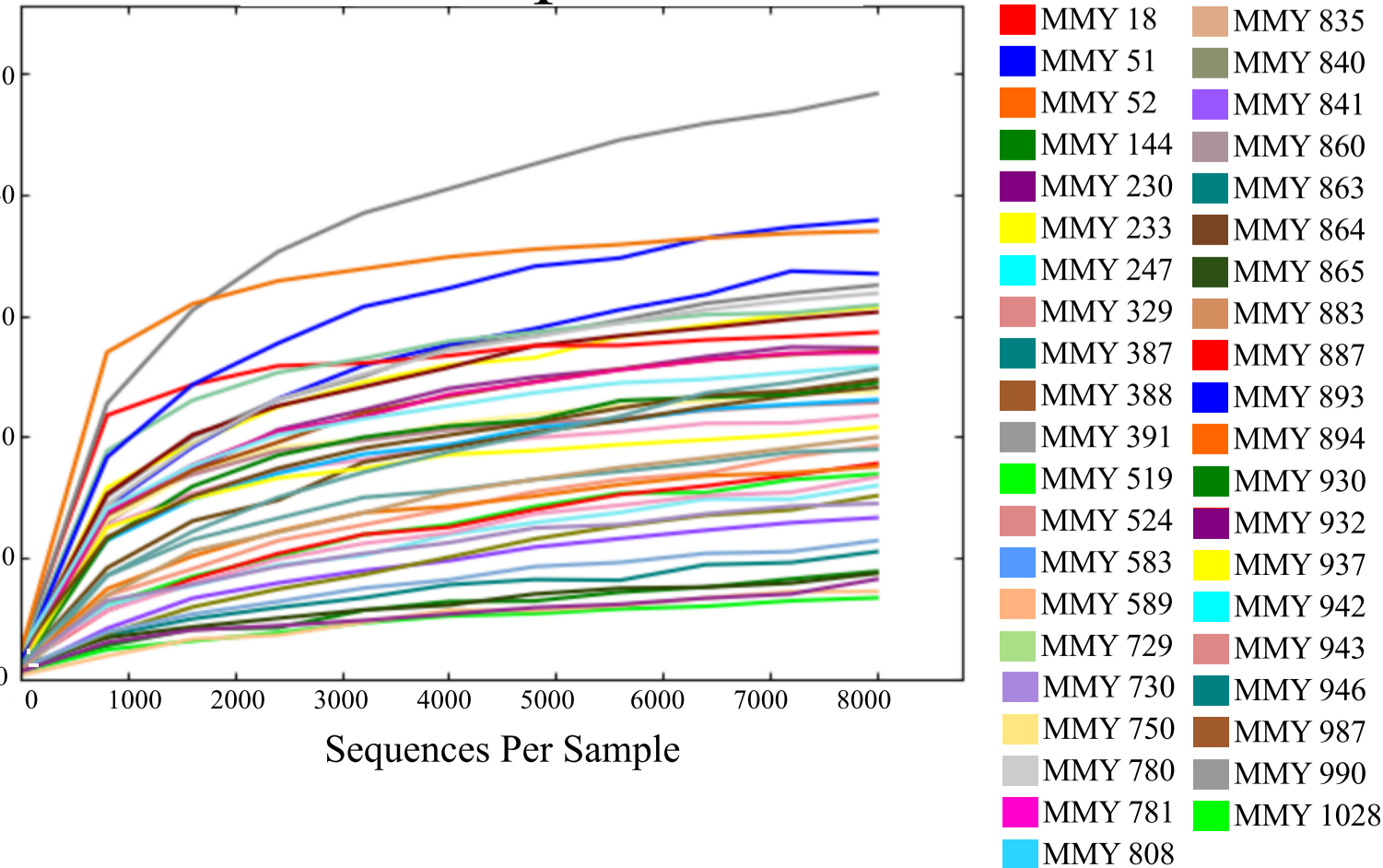

Supplement: Figure S2 — Rarefaction plots for age datasets 1 (A), 3 (B) and 4 (C), in addition to location (D), are displayed. Alpha diversity is measured using phylogenetic diversity (PD). [file peerj-06-4174-s002.pdf]

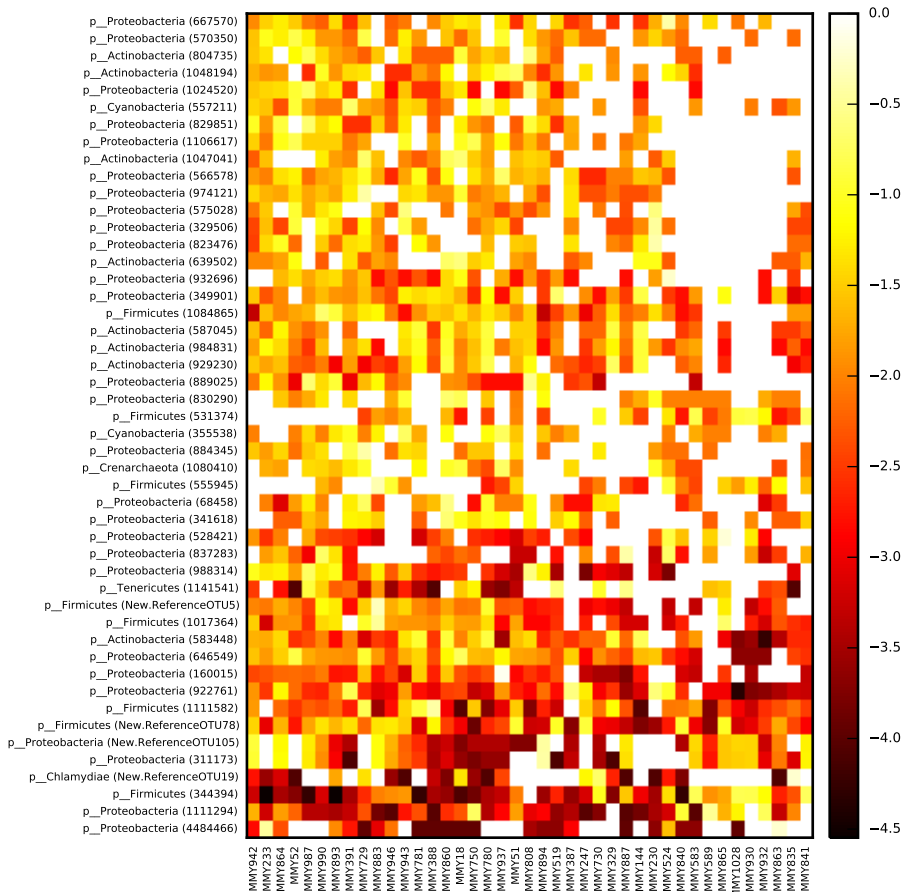

Supplement: Figure S3 — Heatmap displaying the core microbiome across samples (OTUs present in 50% of all samples), highlighting some inter-individual diversity between specific OTUs. [file peerj-06-4174-s003.pdf]

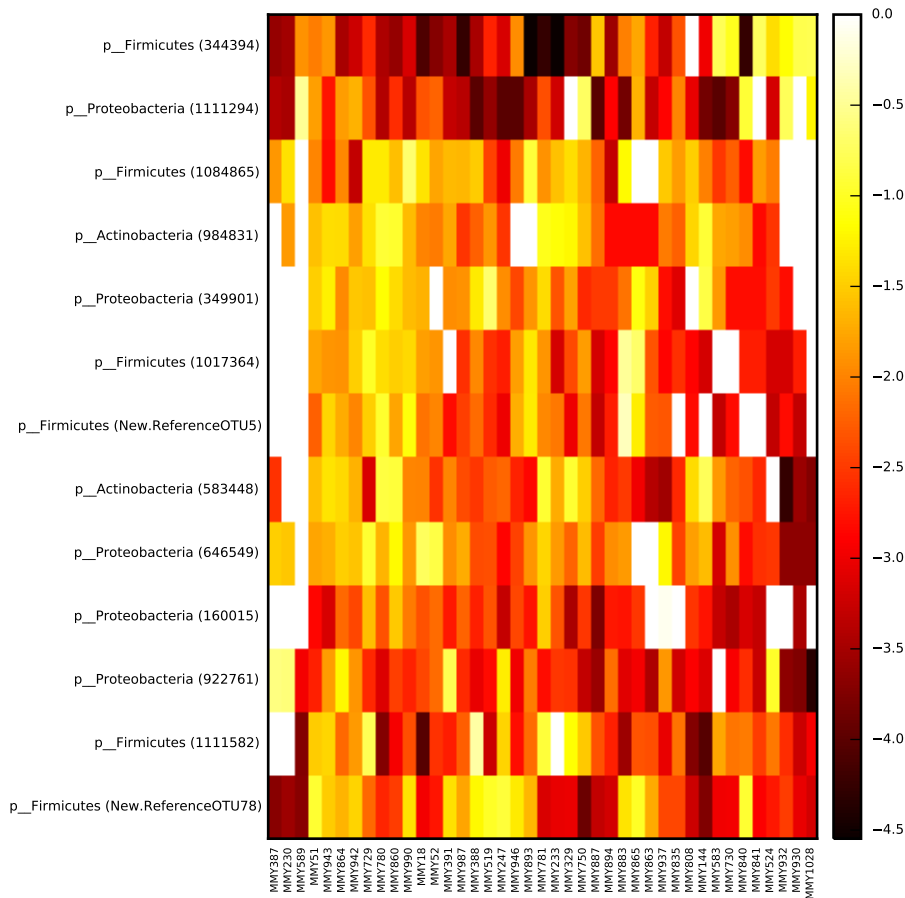

Supplement: Figure S4 — Heatmap displaying OTUs present in the majority (80%) of Myotis myotis samples. [file peerj-06-4174-s004.pdf]

(a)

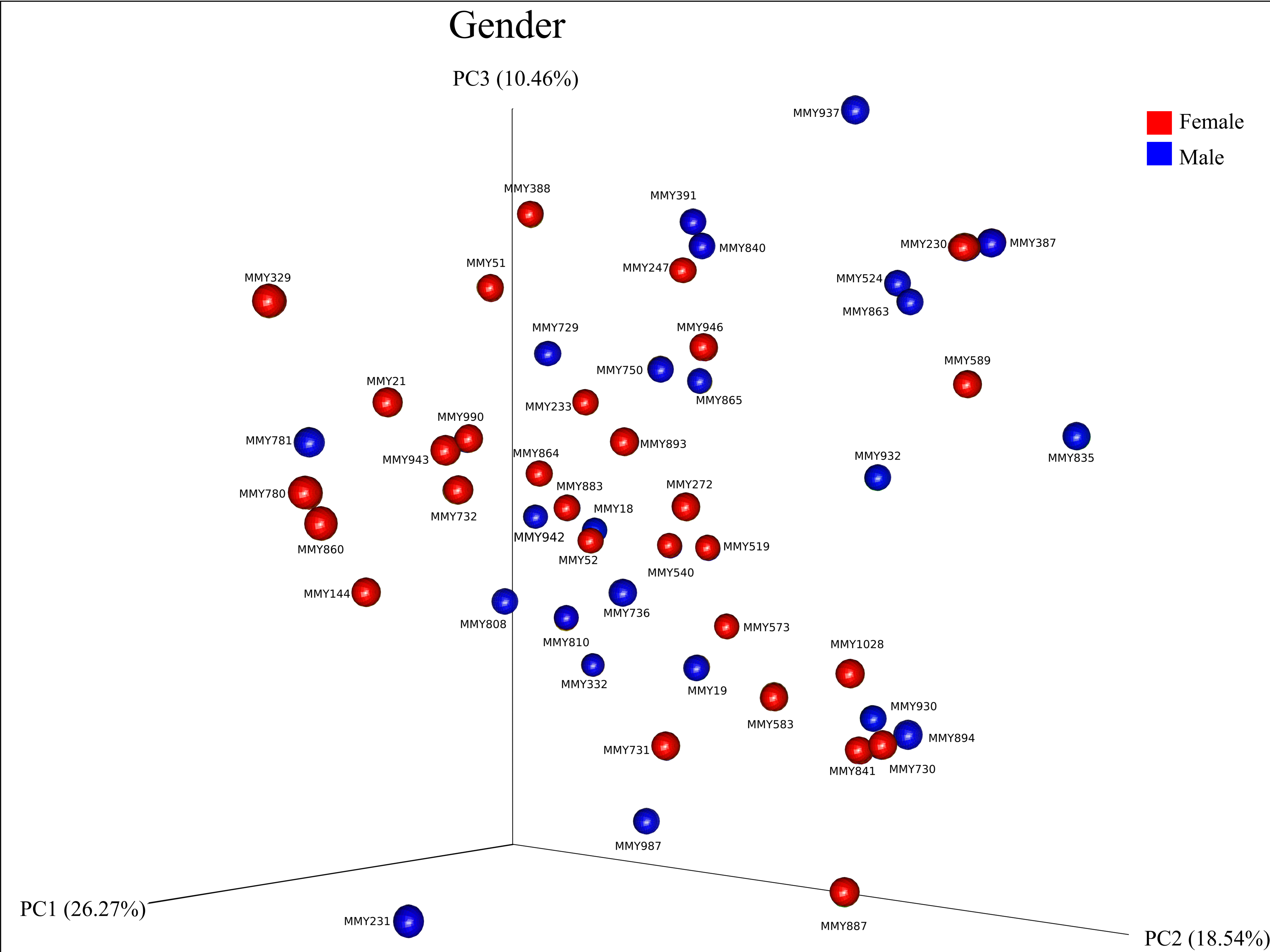

(b)

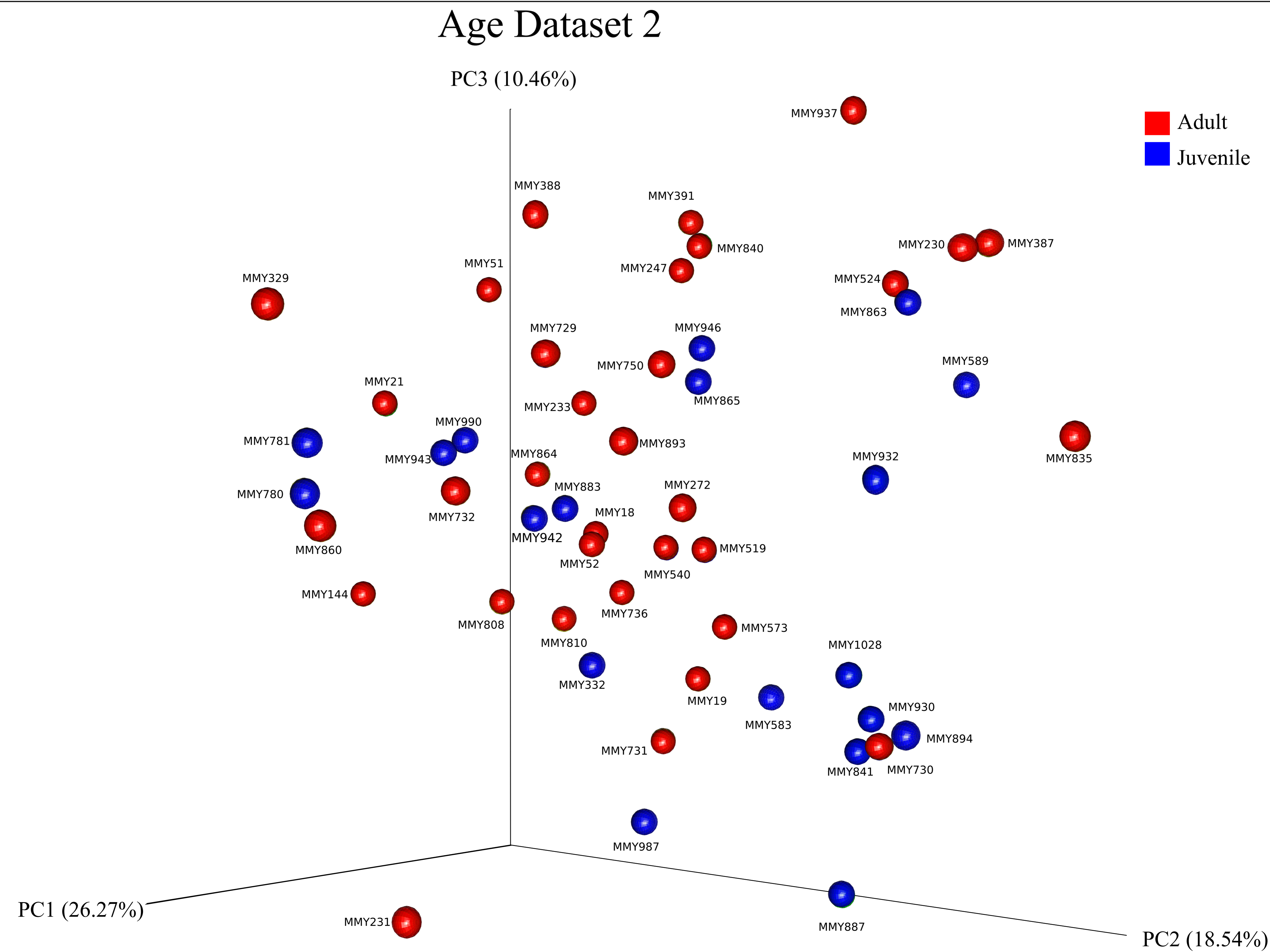

Supplement: Figure S5 — Beta diversity across samples was investigated before rarefaction to determine if any separation of categorical data could be identified. (A) Beta diversity of male and female samples before rarefaction is displayed. (B) Beta diversity using weighted between adults and juveniles in age dataset 2 before rarefaction is displayed. [file peerj-06-4174-s005.pdf]

(a)

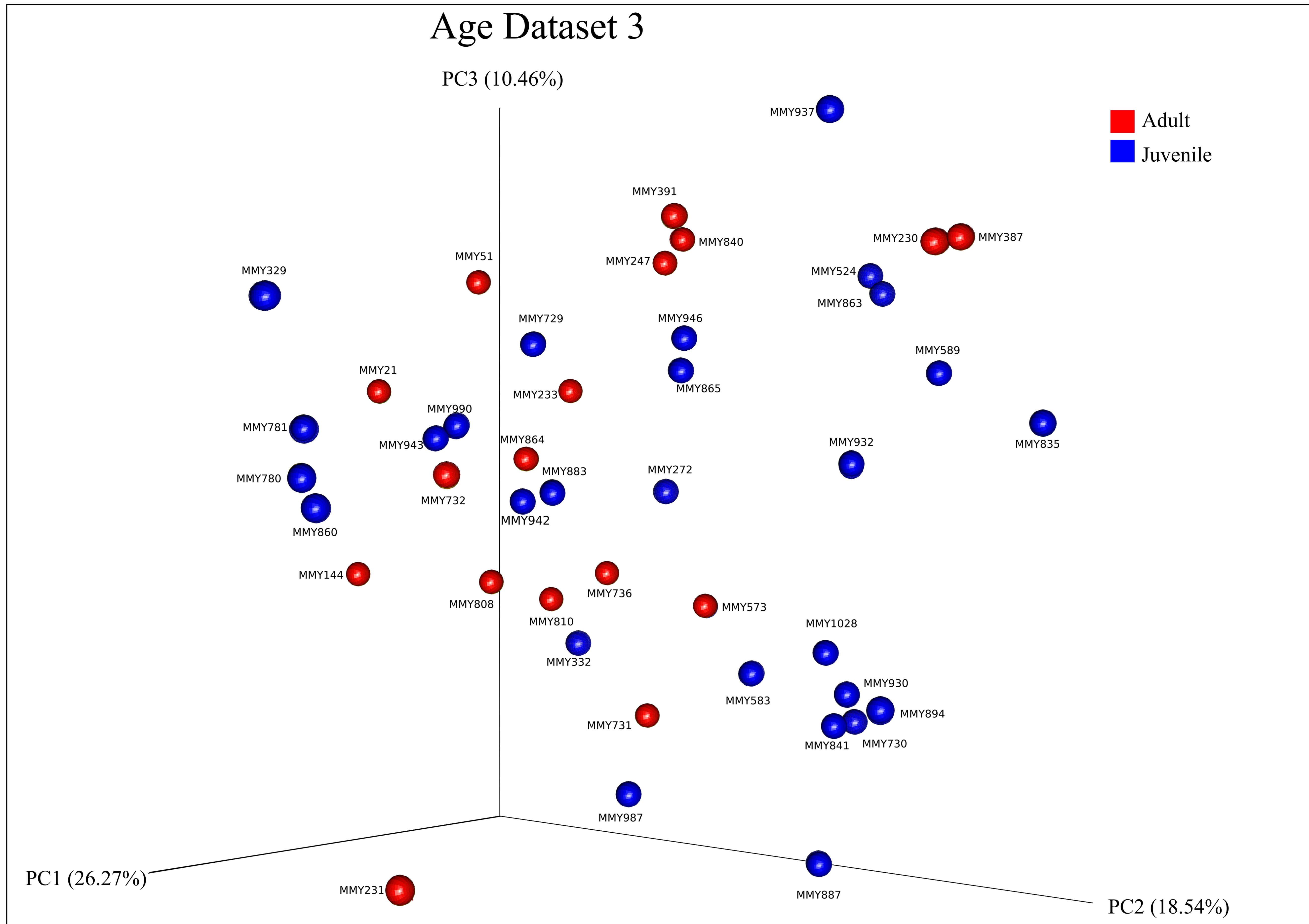

(b)

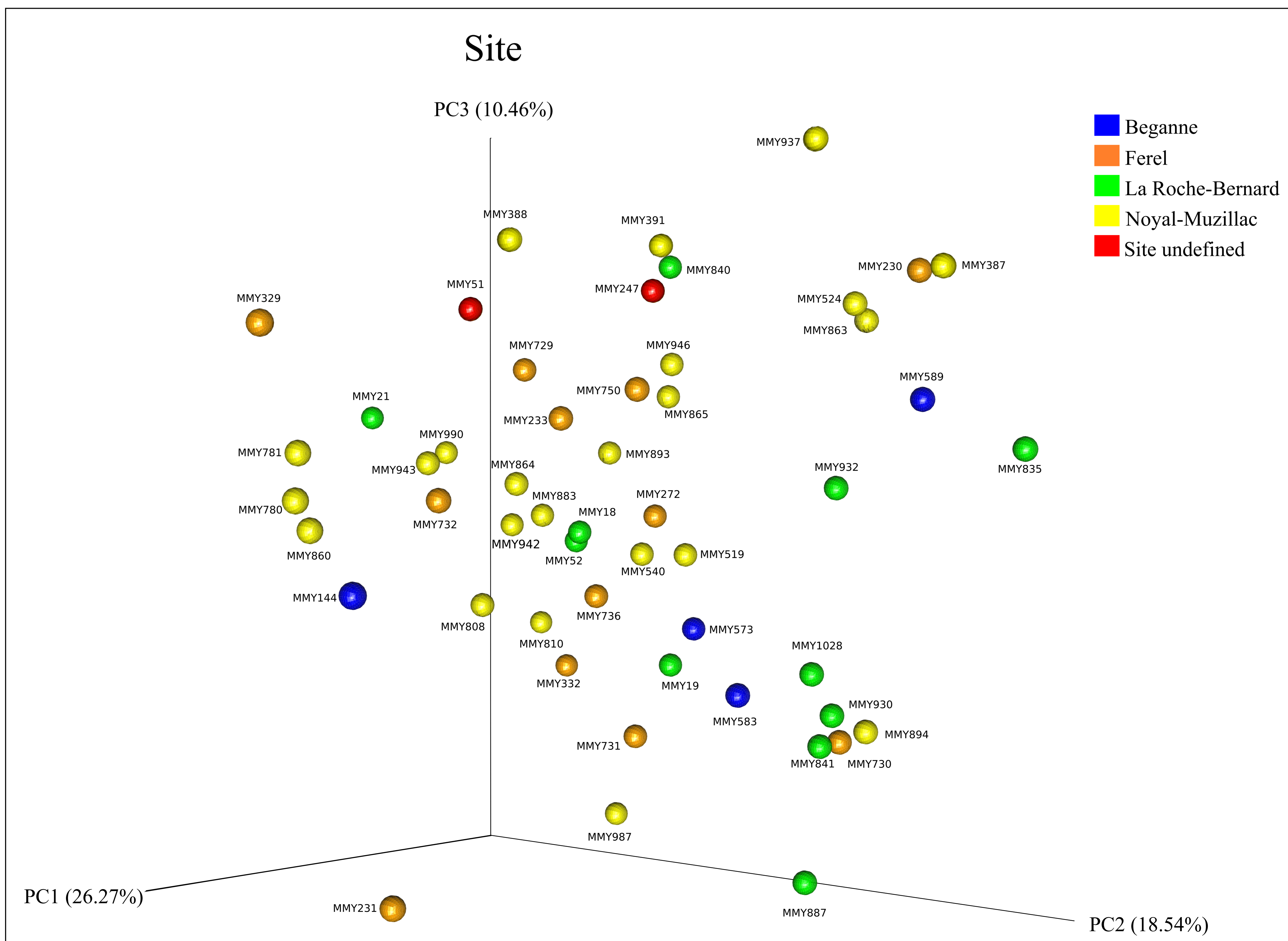

Supplement: Figure S6 — Beta diversity across samples was investigated before rarefaction using PCoA and weighted Unifrac distances for (A) age dataset 3 and (B) sample collection site, representing 55% of variance. [file peerj-06-4174-s006.pdf]

(a)

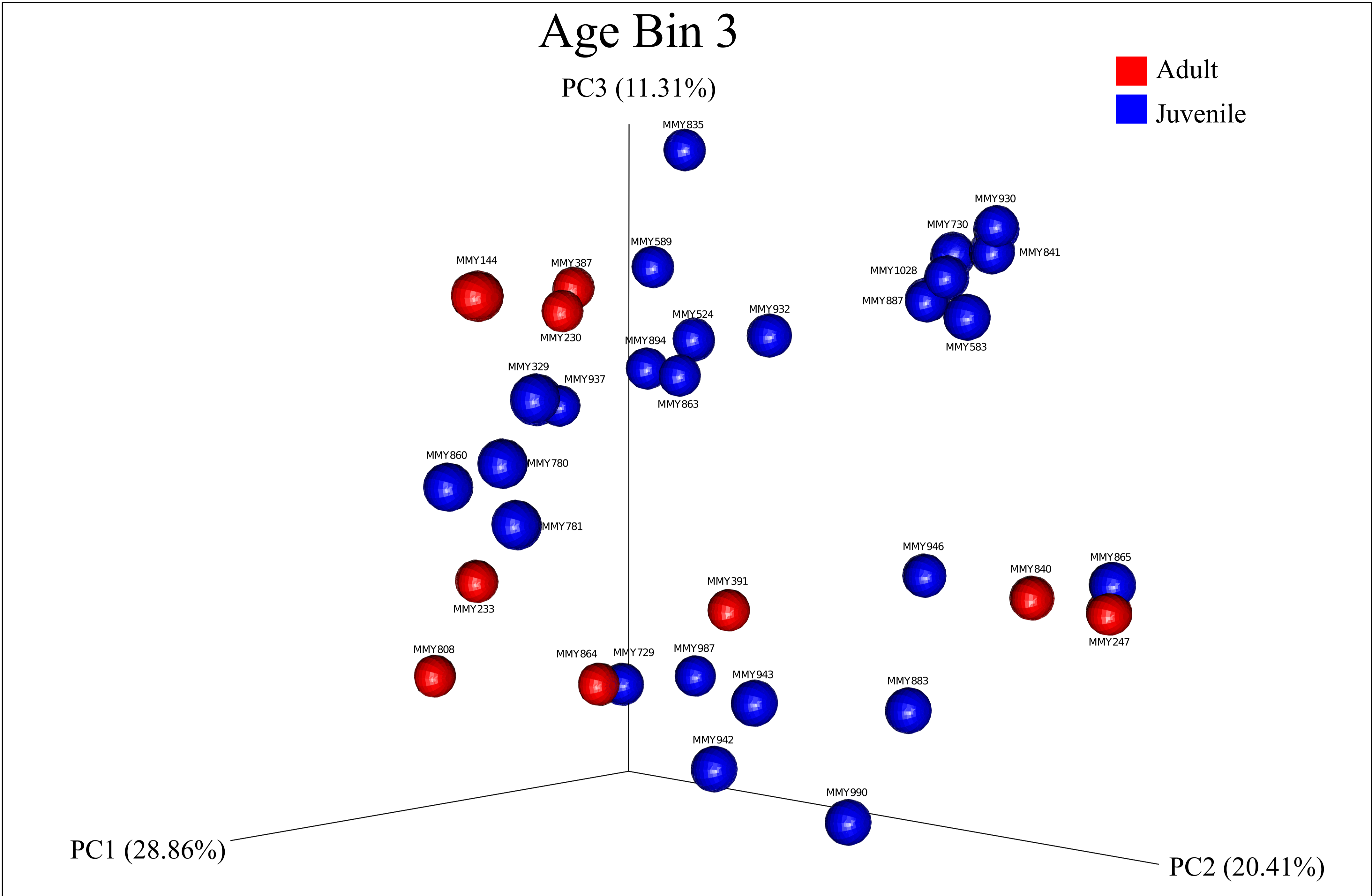

(b)

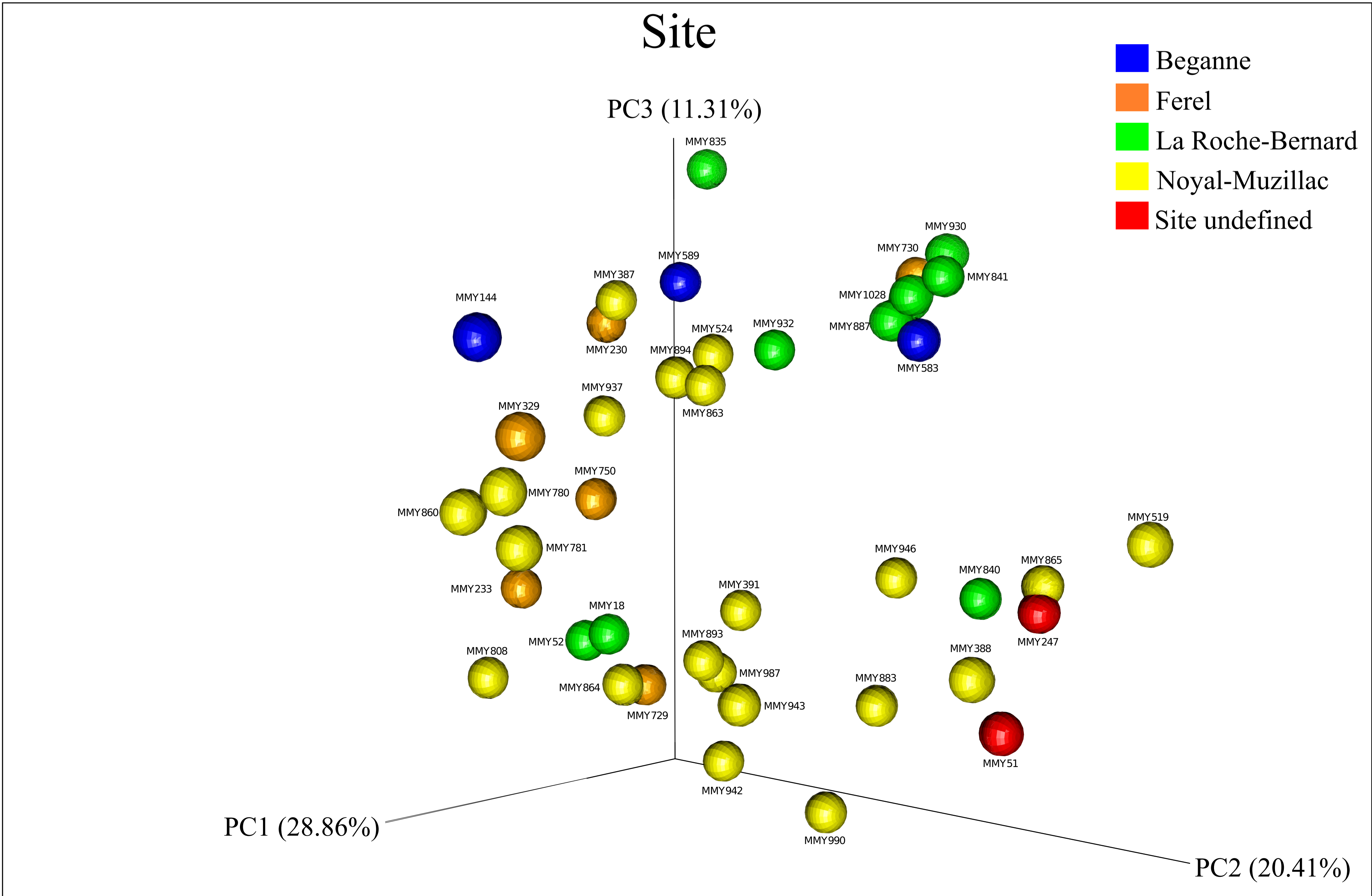

Supplement: Figure S7 — Similarity based on beta-diversity between samples was explored using PCoA and weighted Unifrac distances after rarefaction, explaining 60.59% variance for (A) age dataset 3 and (B) sample collection site. [file peerj-06-4174-s007.pdf]

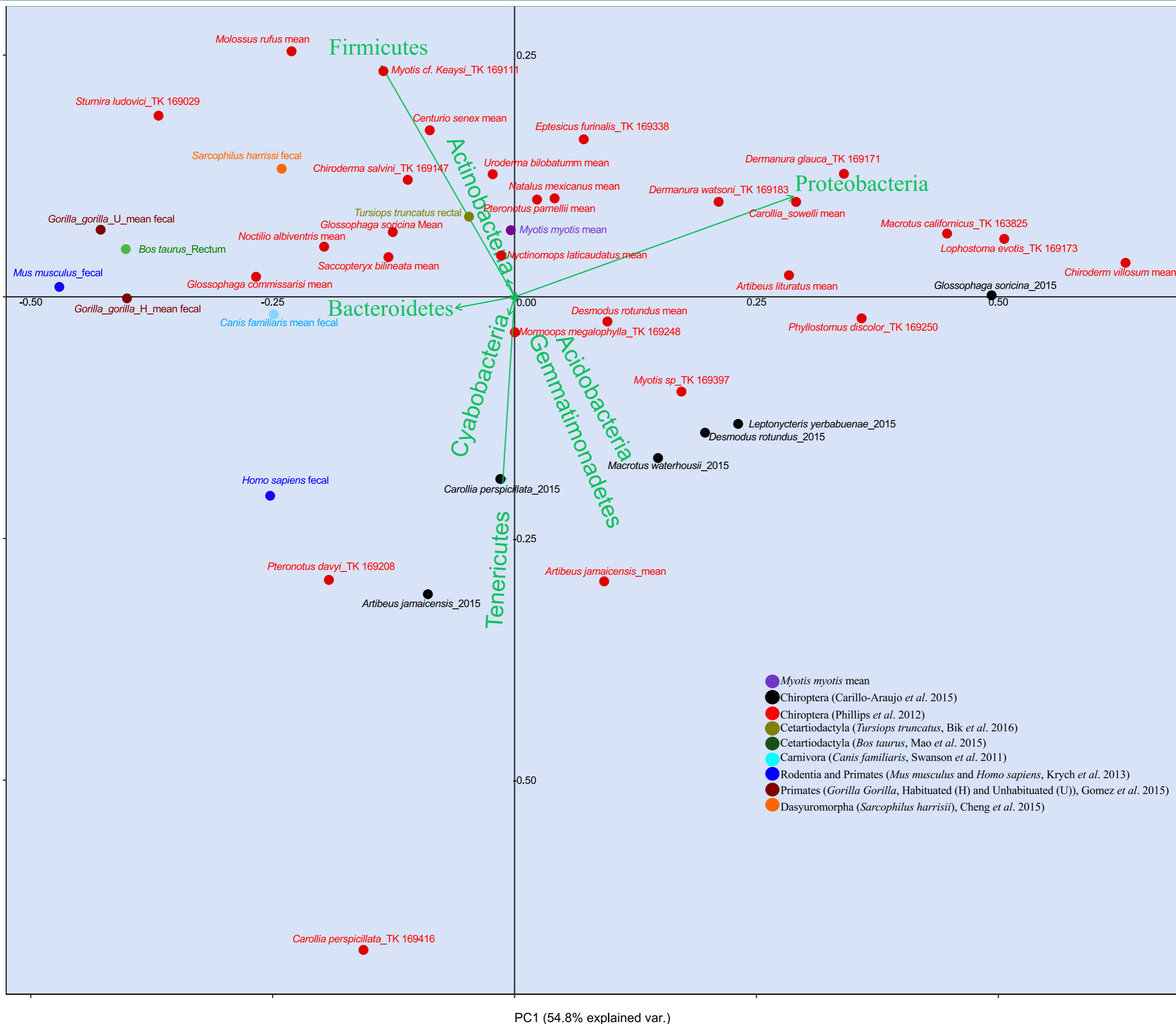

Supplement: Figure S8 — Principal components analyses of nine highly abundance bacteria phyla across mammals, with the first three components displayed, highlighting the differential abundance across various mammalian microbiomes. [file peerj-06-4174-s008.pdf]
